# Zebrafish Xenograft Model for Studying Pancreatic Cancer-Instructed Innate Immune Microenvironment

**DOI:** 10.3390/ijms23126442

**Published:** 2022-06-09

**Authors:** Xue Wang, Wei Li, Haowei Jiang, Cui Ma, Mengling Huang, Xiaona Wei, Wei Wang, Lili Jing

**Affiliations:** Engineering Research Center of Cell & Therapeutic Antibody, Ministry of Education, School of Pharmacy, Shanghai Jiao Tong University, Shanghai 200240, China; anewang@sjtu.edu.cn (X.W.); li-weishen@sjtu.edu.cn (W.L.); haoweijiang@sjtu.edu.cn (H.J.); ma-cui@sjtu.edu.cn (C.M.); morning-huang@sjtu.edu.cn (M.H.); weixiaona@sjtu.edu.cn (X.W.); vivid.sjtu.edu.cn@sjtu.edu.cn (W.W.)

**Keywords:** pancreatic cancer, zebrafish xenograft, innate immune microenvironment, tumor associated myeloid cells

## Abstract

Pancreatic ductal adenocarcinoma (PDAC) has up to half the tumor mass of tumor-associated myeloid cells. Myeloid innate immune cells play important roles in regulating cancer cell recognition and tumor growth. PDAC cells often mold myeloid cells into pro-tumoral state to fuel cancer growth and induce immune suppression. However, how tumor cells educate the innate immune responses remains largely unknown. In this study, we used four different human PDAC cell lines (PANC1, BxPC3, AsPC1, and CFPAC1) to establish the zebrafish xenograft model and investigated the interaction between pancreatic cancer and innate immune cells. The primary tumor-derived cancer cells PANC1 and BxPC3 activated innate immune anti-tumoral responses efficiently, while cancer cells from metastatic tissues AsPC1 and CFPAC1 induced an innate immune suppression and educated innate immune cells towards pro-tumoral state. Chemical conversion of innate immune cells to anti-tumoral state inhibited tumor growth for AsPC1 and CFPAC1. Moreover, genetic and pharmacological inhibition of macrophages also significantly reduced tumor growth, supporting the important roles of macrophages in innate immune suppression. REG4 expression is high in AsPC1 and CFPAC1. Knockdown of REG4 induced innate immune activation and reduced tumor growth in the xenografts, indicating that REG4 is a beneficial target for PDAC therapy. Our study provides a fast in-vivo model to study PDAC-innate immune interaction and their plasticity that could be used to study the related mechanism as well as identify new drugs to enhance immunotherapy.

## 1. Introduction

Pancreatic ductal adenocarcinoma (PDAC), the most common type of pancreatic cancer, is a devastating disease [1]. Despite advances in cancer treatment, the overall 5-year survival rate for PDAC remains less than 8% [2]. Improved strategies such as FOLFIRINOX combined chemotherapy have provided certain amounts of effects, but still show low responses and patients develop resistance quickly [3]. The latest checkpoint-based immunotherapies have also shown poor therapeutic outcomes in patients with PDAC [4].

The tumor microenvironment (TME) of PDAC plays an essential role in cancer progression and largely determines the outcome of various therapies [5]. The PDAC TME is characterized by dense fibrotic stroma and extensive infiltration of myeloid cells that could represent up to a half of the tumor mass [6,7]. The myeloid cells are a group of innate immune cells including monocytes, macrophages, granulocytes, and dendritic cells (DCs). They play important roles in cancer cell recognition, initiation of inflammation, and anti-tumor responses [8]. However, tumor cells also develop mechanisms to evade immune surveillance and could mold myeloid cells to serve the tumor. Functionally, tumor-associated myeloid cells are often divided into anti- or pro-tumor subgroups [9,10,11]. The anti-tumoral group are associated with immunostimulatory cells with the secretion of pro-inflammatory cytokines (e.g TNF-α, IL-12, and IL-6). The pro-tumor cells are considered immunoregulatory cells that produce anti-inflammatory cytokines (e.g IL-10 and TGF-b). As PDAC cancer progresses, tumor educates innate immune cells into a pro-tumoral state that supports immune evasion, disease progression, and metastasis. Myeloid cells are recognized as the key drivers for the immune-suppressive microenvironment for PDAC and negatively impact standard therapies [12,13]. To improve the therapeutic response in PDAC, it is necessary to understand the interaction of innate immune cells and PDAC and their potential impacts.

Macrophages are the most abundant infiltrated myeloid innate immune populations. and are activated according to specific environmental stimuli. Tumor-associated macrophages (TAMs) exhibit phenotypic heterogeneity ranging from tumor-inhibiting M1-like to tumor-promoting M2-like [14]. In PDAC, most TAMs display an M2-like phenotype [12,15]. M2 macrophages secrete anti-inflammatory cytokines and render an immunosuppressive environment promoting oncogenic progression [16,17]. Recent studies demonstrate that pharmacological targeting of macrophages or reprogramming of M2-like TAM to M1 state could modulate the innate immune-suppressive response, abrogate tumor growth, and partially overcome the insensitivity and drug resistance for pancreatic cancer [18,19,20]. These studies support the idea that the macrophage is a promising target for PDAC therapy. Neutrophils are another prominent innate immune cell type in the PDAC TME [9,11]. Although the exact roles of neutrophils in PDAC are not well characterized, neutrophils have also been suggested to show different activation status from an immunostimulatory (N1-like) type to immunosuppressive (N2-like) type, similar to macrophages [9]. It is known that macrophages could interact with neutrophils together to induce an innate and adaptive immunosuppressive TME. However, how PDAC tumor instructs TAMs to an M2-like state and the innate immune pro-tumoral state remains largely unknown. Identifying the responsible molecular mechanisms will help to discover novel therapies for treating pancreatic cancer. 

The transgenic or tumor xenograft mouse models are widely used to study immune–tumor cell interactions. However, the use of mouse models has some limits such as high costs, is time-consuming, and has the potential requirement of immunosuppressed strains [21]. Recently, zebrafish have been increasingly used for cancer research [22]. It shows a high degree of physiological and genetic similarity to mammals and can be used to mimic the tumor microenvironment. Zebrafish xenotransplantation model is widely used to evaluate cancer progression, neo-angiogenesis, and drug response/resistance [23,24,25]. In zebrafish, the innate and adaptive immune system is highly conserved [26], and the cancer cells and innate immune cells can be fluorescently labelled and directly observed in the living animal due to optical clarity of zebrafish larvae. In addition, the full maturation of adaptive immunity only occurs at 2–3 weeks post-fertilization and the transplantation can be easily performed without the need for immunosuppressive drugs or immunocompromised variants. Here, we set up a zebrafish xenograft model of pancreatic cancer to study the crosstalk between cancer and innate immune cells. Our results demonstrate that zebrafish could read immune signaling from pancreatic cancers, modulate innate immune cells, particularly macrophages towards different phenotypes, and establish a distinct microenvironment according to different cancers in just 4 days. Thus, it is a novel research tool for investigating the crosstalk between pancreatic tumor and myeloid innate immune cells.

## 2. Results

### 2.1. The Pancreatic Cancer Cells Show Differential Engraftment Profile in Zebrafish Xenografts

**Figure 1:**
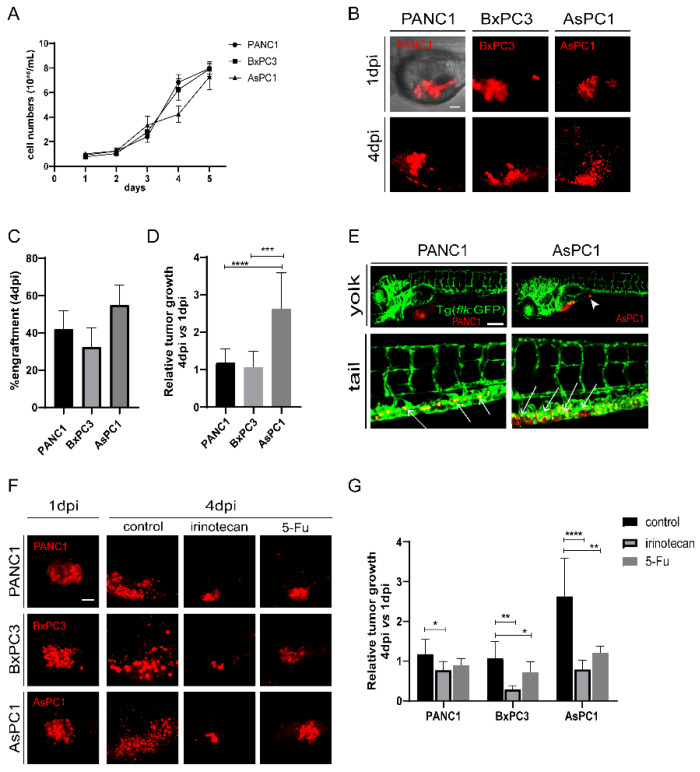
The pancreatic cancer cells show differential engraftment profile in zebrafish. (**A**) The growth curve of in-vitro cultured PANC1, BxPC3, and AsPC1 cells. (**B**) Representative confocal images of PANC1, BxPC1, and AsPC1 xenografts at 1 dpi and 4 dpi. (**C**) Engraftment quantification for PANC1, BxPC1, and AsPC1 cells at 4 dpi. Results are shown as means ± SEM from 50 different individuals. (**D**) Relative tumor growth (4 dpi vs. 1 dpi) for PANC1, BxPC3, and AsPC1 xenografts. Results are from nine different individuals (*** *p* < 0.001, **** *p* < 0.0001, *t* test). (**E**) PANC1 and AsPC1 showed metastasis in the intestine (white arrowheads) and in the caudal hematopoietic tissue (white arrows). (**F**) Representative confocal images of PANC1, BxPC3, and AsPC1 xenografts at 1 dpi or at 4 dpi after DMSO (control), irinotecan (50 μM) or 5-Fu (2 mM) treatment. (**G**) Relative tumor growth (4 dpi vs. 1 dpi) for PANC1, BxPC3, and AsPC1 xenografts after treatment with DMSO, irinotecan or 5-Fu. Results are from nine different individuals (* *p* < 0.05, ** *p* < 0.01, ***p < 0.001, **** *p* < 0.0001, *t* test). dpi, days post injection. Scale bars in (**B**,**F**), 50 µm; in (**E**), 75 µm.

Since cancer cells significantly affect the TME, we first selected three human pancreatic cancer cell lines, PANC1 and BxPC3 (both derived from the primary tumors of PDAC patients), and AsPC1 (a pancreatic cancer cell line derived from metastatic ascites) as sources for xenografting. The three cell lines did not show a significant difference in growth speed in vitro, with AsPC1 growing slightly slower than the other two cell lines (Figure 1A). The three cells were then fluorescently labeled with a lipophilic dye (Dil) and injected into the yolk sac of 48 hour-post-fertilization (hpf) zebrafish embryos independently to establish zebrafish xenografts (Figure 1B). To check the progression of xenografted cancer cells in vivo, we recorded and analyzed the zebrafish larva under fluorescence microscopy daily (Figure S1A). At 4 days post injection (dpi), all three cell lines were efficiently engrafted in zebrafish embryos, but AsPC1 presented a higher engraftment rate (54.8 ± 10.9%) than PANC1 (42.1 ± 9.7%) and BxPC3 (32.5 ± 10.1%) (Figure 1C and Figure S1A). We compared the relative tumor size in the xenografts at 4 dpi with respect to 1 dpi, and found that AsPC1 tumor grew significantly larger than PANC1 and BxPC3 cells (AsPC1:262.9 ± 75.1%; PANC1:118.0 ± 36.7%, and BxPC3:107 ± 42.0%, Figure 1D), distinctly from the cancer cell growth in vitro.

In a group of embryos xenografted with PANC1 or AsPC1 tumors, we detected cancer cell clusters in the intestine and the caudal hematopoietic tissue (CHT) region at 4 dpi (Figure 1E), indicating the metastatic potential of these two cell lines. In contrast, BxPC3 showed limited dissemination, in agreement with a previous study that shows that BxPC3 cells do not proliferate and disseminate well in zebrafish xenografts [27]. We also examined the angiogenesis of the three tumors. To do that, the three pancreatic cancer cell lines were transplanted into Tg (*flk:GFP*) zebrafish with GFP-labeled vasculature [28]. We found that PANC1, BxPC3, and AsPC1 were all able to recruit blood vessels from the zebrafish host, stimulate angiogenesis from the subintestinal vein (SIV) plexus, and form a dense vessel network in the tumor area at 5 dpi (Figure S1B).

To test whether zebrafish xenografts could respond to chemotherapies, we assessed the impact of irinotecan and 5-Fu on the tumor cell growth. Irinotecan and 5-Fu inhibited the proliferation of PANC1, BxPC3, and AsPC1 cells in vitro similarly in a dose-dependent manner (Figure S2A–C and data not shown). To test the effects on zebrafish xenografts, we selected a high and tolerated concentration for each compound that does not cause visible abnormalities in embryos. All xenografts at 24 hpi (hours post injection) were randomly distributed, and treated with control, irinotecan or 5-Fu for consecutive 3 days (Figure 1F). Irinotecan treatment induced a significant reduction of tumor size for all tumors, and 5-Fu induced a significant tumor reduction for BxPC3 and AsPC1. Interestingly, AsPC1 is much more sensitive to both irinotecan and 5-Fu treatment than PANC1 and BxPC3 (Figure 1G). Together, our results show that three human pancreatic cancer cell lines were able to be transplanted in zebrafish larva, maintained their angiogenic and metastatic potential, and that they presented different proliferation dynamics and sensitivities to chemotherapy in vivo, significantly different from their performance in vitro.

### 2.2. Pancreatic Cancer Cells Induce Distinct Innate Immune Responses in Zebrafish Xenografts

**Figure 2:**
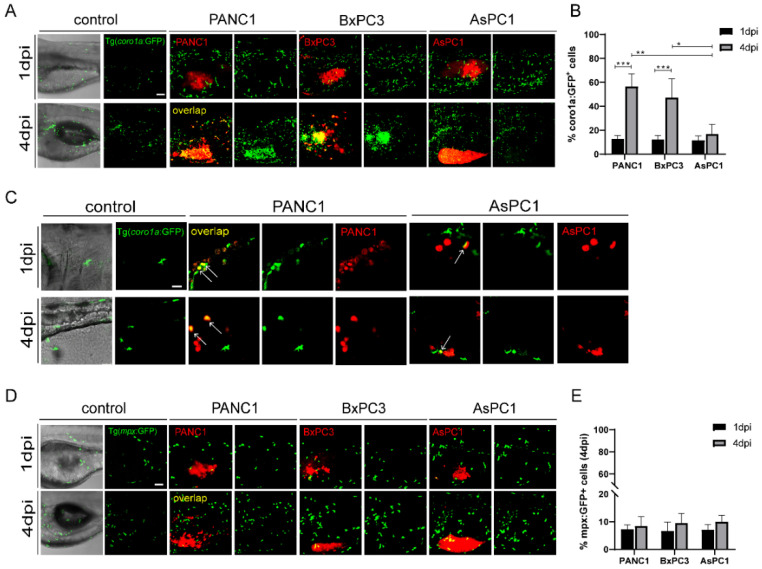
The pancreatic cancer cells show distinct interaction with the innate immune cells in zebrafish. (**A**) Representative confocal images of Tg(*coro1a: GFP*)-labeled innate immune cells in PANC1, BxPC3, and AsPC1 xenografts at 1 dpi and 4 dpi. (**B**) Qualification of innate immune cell percentage in PANC1, BxPC3, and AsPC1 cells at 1 dpi and 4 dpi (no. of innate immune cells/no. of tumor cells × 100). Results are shown as means ± SEM from nine different individuals (* *p* < 0.05, ** *p* < 0.01, *** *p* < 0.001, ANOVA). (**C**) Enlarged confocal images of innate immune cells co-localized with PANC1 and AsPC1 xenografts at 1 dpi and 4 dpi. Arrows point to the co-localized cells. (**D**) Representative confocal images of Tg(*mpx: GFP*)-labeled neutrophils in PANC1, BxPC3, and AsPC1 xenografts at 1 dpi and 4 dpi. (**E**) Qualification of neutrophils percentage in PANC1, BxPC3, and AsPC1 cells at 1 dpi and 4 dpi (no. of neutrophils/no. of tumor cells × 100). Results are from nine different individuals. dpi, days post injection. Scale bars in (**A**,**D**), 50 µm; in (**C**), 25 µm.

Next, we examined the interaction of the three cancer cells with the innate immune cells in zebrafish xenografts. To do that, we injected PANC1, BxPC3, and AsPC1 cells into Tg(*coro1a: GFP*) zebrafish hosts, which labels the innate immune cells including neutrophils and macrophages, and also marks the lymphocytes in the thymus [29]. We noted that PANC1 and BxPC3 tumors recruited a significant higher number of innate cells to the TME at 4 dpi compared to 1 dpi. In contrast, AsPC1 cells recruited significantly fewer immune cells (Figure 2A). The co-localization of innate immune cells with PANC1 or BxPC3 cells was strongly increased from 1 dpi to 4 dpi, while the co-localization remained unchanged with AsPC1 cells (Figure 2B). Many *coro1a: GFP^+^* immune cells that are co-localized with PANC1 have rounded cellular morphology with fewer protrusion (Figure 2C), which are characteristics of a phagocytotic cell as previously suggested [30]. Thus, *coro1a: GFP^+^* immune cells are likely active and probably phagocytosing the tumor cells. These data together indicate that AsPC1 is less immunogenetic compared to PANC1 and BxPC3 and has an ability to evade innate immune detection in the xenografts. 

Next, we used Tg(*mpeg1: GFP*) to visualize the macrophage reaction to different cancer cells [8]. However, due to the weak fluorescence, we barely detected green-colored macrophages in zebrafish xenografts. We then used Tg(*mpx: GFP*) to visualize the behavior of neutrophils [31]. At 4dpi, PANC1, BxPC3, and AsPC1 all recruited comparable neutrophils to the tumor, and to a much lesser degree compared to *coro1a: GFP^+^* cells. In addition, the co-localization of neutrophils with cancer cells was not changed from 1 dpi to 4 dpi for all three cell lines (Figure 2D,E). Comparing the different responses of *coro1a: GFP^+^* and *mpx: GFP*^+^ cells in in zebrafish xenografts, we hypothesize that macrophages might be the major population that quickly respond to the pancreatic cancer cells in the TME.

### 2.3. Pancreatic Cancer Cells Modulate Innate Immune Cells to Pro-Tumor or Anti-Tumor States

**Figure 3:**
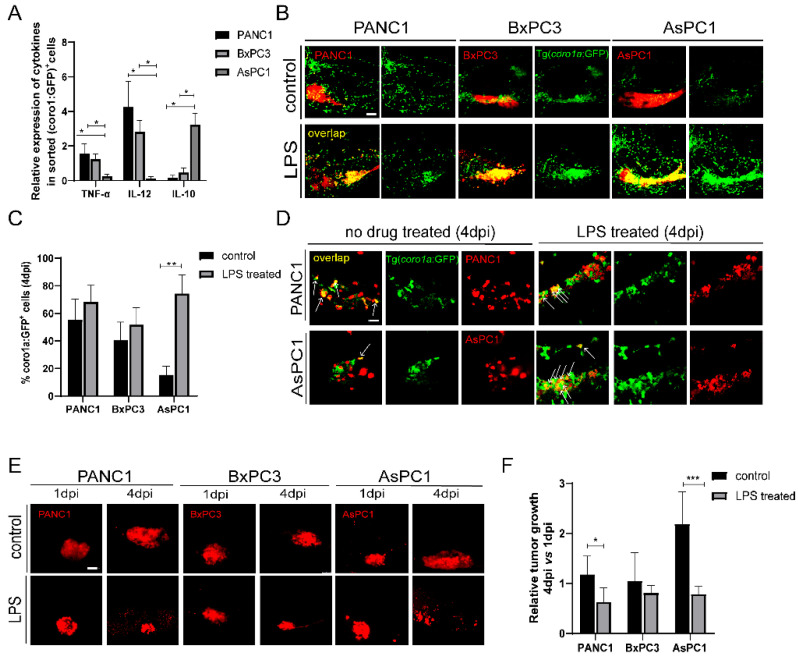
PANC1, BxPC3, and AsPC1 cells induce anti-tumoral or pro-tumoral state of innate immune cells. (**A**) The relative expression of TNF-α, IL-12, and IL-10 in *coro1a: GFP^+^* cells sorted from PANC1, BxPC3, and AsPC1 xenografts (* *p* < 0.05, *t* test). (**B**) Representative confocal images of innate immune cells in PANC1, BxPC3, and AsPC1 xenografts at 4 dpi after treatment with DMSO (control) or LPS (150 μg/mL). (**C**) Qualification of *coro1a: GFP*^+^ innate immune cell percentage in PANC1, BxPC3, and AsPC1 cells at 4 dpi under the control and LPS condition (no. of innate immune cells/no. of tumor cells × 100). Results are shown as means ± SEM from nine different individuals (** *p* < 0.01, *t* test). (**D**) Enlarged confocal images of innate immune cells co-localized with PANC1 and AsPC1 cells at 4 dpi under the control or LPS treatment. Arrowheads point to the co-localized cells. (**E**) Representative confocal images of PANC1, BxPC3, and AsPC1 xenografts at 1 dpi and 4 dpi with the treatment of DMSO (control) or LPS (150 μg/mL) respectively. (**F**) Relative tumor growth in xenografts (4 dpi vs. 1 dpi) after being treated with DMSO or LPS. Results are from nine different individuals (* *p* < 0.05, ** *p* < 0.01, *** *p* < 0.001, *t* test). dpi, days post injection. Scale bars in (**B**,**E**), 50 µm; in (**D**), 25 µm.

The transplanted cancer cells could instruct innate immune cells to pro-tumoral and anti-tumoral states that produce distinct cytokines. We examined the cytokine expression of *coro1a: GFP^+^* cells in PANC1, BxPC3, and AsPC1 xenografted larvae. We sorted *coro1a: GFP^+^* cells from the xenografts at 4 dpi and examined the representative cytokine expression by quantitative PCR (qPCR). Compared to PANC1 and BxPC3 tumor, *coro1a: GFP^+^* cells from AsPC1 xenografts expressed a low level of TNF-α and IL-12, and a higher level of IL-10 (Figure 3A). These results support the idea that AsPC1 cells might induce innate immune cells to a pro-tumoral state, while PANC1 and BxPC3 induce an anti-tumoral state. Previous reports have demonstrated that lipopolysaccharide (LPS) can activate innate immune cells into an anti-tumoral state in zebrafish [32,33]. We then used LPS to modulate the innate immune cell states in the xenografts. LPS treatment led to a significant increase of co-localization of innate immune cells with PANC1, BxPC3, and AsPC1 cells, particularly for AsPC1 (Figure 3B,C). We also noted that an increased phagocytosis of AsPC1 cells by immune cells after LPS induction (Figure 3D). LPS did not influence the growth of PANC1, BxPC3, and AsPC1 cells in vitro at various concentrations (Figure S3). After 3 days of LPS treatment in transplanted larvae, PANC1 and BxPC3 presented an average 20–30% decrease in the tumor size, while AsPC1 showed a remarkable 60% tumor reduction (*p* < 0.001, Figure 3E,F). These results together supported that AsPC1 likely induced innate immune cells into pro-tumor state to promote tumor growth, and LPS treatment activated innate immune cells and regressed tumor growth.

### 2.4. Innate Immune Cells, Particularly Macrophages, Modulate Pancreatic Tumor Progression in Zebrafish Xenografts

**Figure 4:**
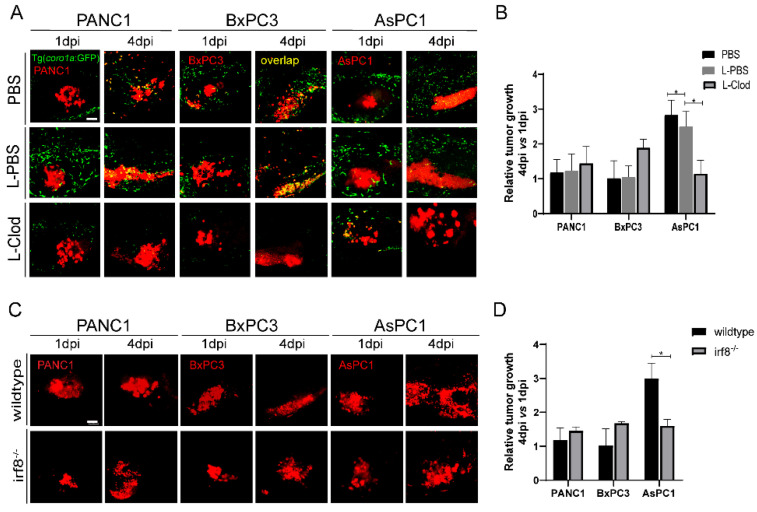
Zebrafish innate immune cells regulate PANC1, BxPC3, and AsPC1 tumor progression. (**A**) Representative confocal images of PANC1, BxPC3, and AsPC1 xenografts in embryos co-injected with PBS, L-PBS or liposome-clodronate (L-clodronate) at 1 dpi and 4 dpi. (**B**) Relative tumor cell growth for PANC1, BxPC3, and AsPC1 in PBS, L-PBS, or L-clodronate-treated groups. Results are from nine different individuals (* *p* < 0.05, *t* test). (**C**) Representative confocal images of PANC1, BxPC3, and AsPC1 xenografts in WT and *irf8^−/−^* mutant embryos at 1 dpi and 4 dpi. (**D**) Relative tumor growth of PANC1, BxPC3, and AsPC1 in WT and *irf8^−/−^* mutant embryos. Results are shown as means ± SEM from nine different individuals (* *p* < 0.05, *t* test). dpi, days post injection. Scale bar, 50 µm.

We next examined the direct function of innate immune cells in tumor growth. Since macrophages likely play important roles in our system, we used the liposome-clodronate (L-clodronate) to primarily deplete macrophage in zebrafish larvae as used in previous studies [26,34]. We observed a strong reduction of *coro1a: GFP^+^* innate immune cells particularly in the TME (Figure 4A), supporting the fac that these cells are likely macrophages. More importantly, with the treatment of L-clodronate, the tumor sizes of PANC1 and BxPC3 were slightly increased, but AsPC1 tumor size was decreased by 70% (Figure 4B).

Next, we implanted three pancreatic cancer cells into *irf8^−/−^* zebrafish that has a strong reduction of macrophages and an increase of neutrophils with no change of total primitive myeloid cells number [35,36,37,38]. The results showed that the tumor sizes for PANC1 and BxPC3 in *irf8^−/−^* were slightly increased. In contrast, in *irf8^−/−^* mutants, AsPC1 showed a significant 50% decrease of the tumor size (Figure 4C,D). These results suggest that AsPC1 not just suppressed the host innate immune system, but likely hijacked the innate immune cells, particularly macrophages, to promote tumor growth.

To further verify the function of macrophages in AsPC1 tumor progression, we used the chemotherapy drug paclitaxel to treat the xenografts. It was shown before that paclitaxel suppresses the induction of M2 macrophages at low doses that do not affect the cancer cell proliferation [39]. Previous studies have also shown that in-vitro cultured PANC1, BxPC1, and AsPC1 cells have almost the same sensitivity towards paclitaxel. We treated the zebrafish xenografts with paclitaxel at a low dose, which did not affect the tumor size for PANC1 and BxPC3. In contrast, the same paclitaxel treatment induced a significant reduction in tumor size for AsPC1 (Figure S4A,B). These data support the idea that low-concentration paclitaxel treatment only inhibited AsPC1 tumor growth likely through suppressing the polarization of M2 macrophages and the pro-tumoral state of innate immune cells in AsPC1 xenografts.

### 2.5. A Second Metastatic Pancreatic Cancer May also Utilize Pro-Tumoral Innate Immune Cells to Support Tumor Growth

**Figure 5:**
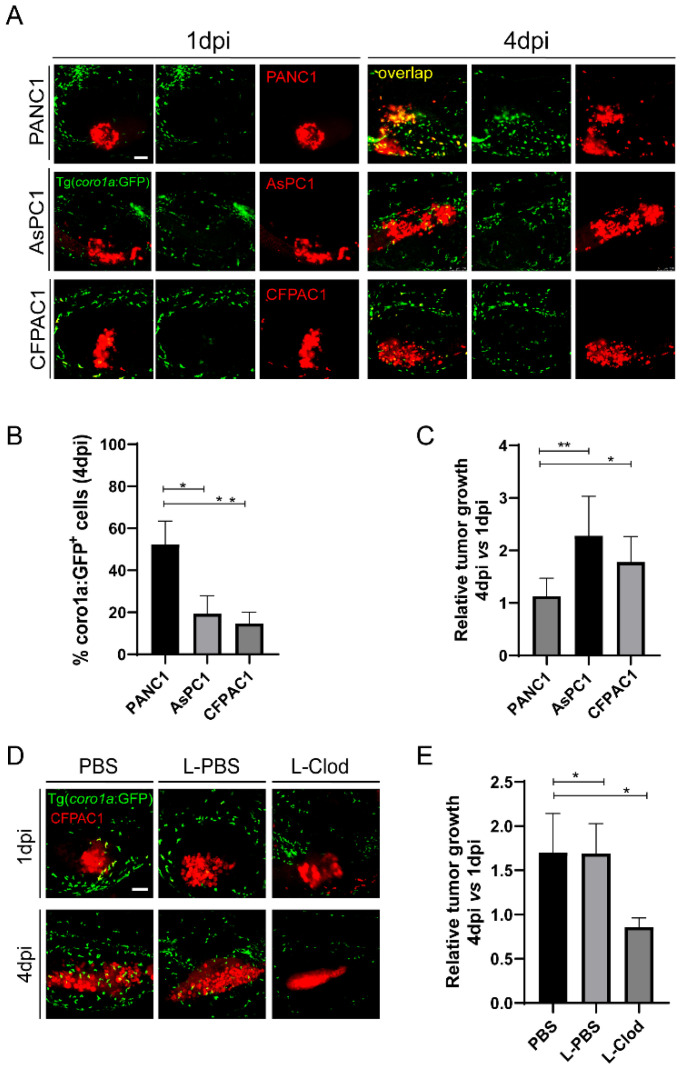
CFPAC1 also hijacks innate immune cells to promote tumor growth in zebrafish. (**A**) Representative confocal images of PANC1, AsPC1, and CFPAC1 xenografts at 1 dpi and 4 dpi. (**B**) Qualification of *coro1a: GFP*^+^ innate immune cell percentage in PANC1, AsPC1, and CFPAC1 cells at 4 dpi (no. of innate immune cells/no. of tumor cells × 100). Results are shown as means ± SEM from nine different individuals (* *p* < 0.05, ** *p* < 0.01, *t* test). (**C**) Relative tumor growth of PANC1, AsPC1, and CFPAC1. Results are from nine different individuals (* *p* < 0.05, ** *p* < 0.01, *t* test). (**D**) Representative confocal images of CFPAC1 xenografts in the embryos co-injected with PBS, L-PBS or liposome-clodronate (L-clodronate) at 1 dpi and 4 dpi. (**E**) Relative tumor growth of CFPAC1 in PBS-, L-PBS- or L-clodronate-treated groups. Results are from nine different individuals (* *p* < 0.05, *t* test). dpi, days post injection. Scale bar, 50 µm.

Both PANC1 and BxPC3 are primary tumor cell lines, while AsPC1 is a cell line from the metastatic tumor [40]. We hypothesize that metastatic pancreatic cancer cells might induce the innate pro-tumoral state more robustly in zebrafish xenograft. To do that, we used another pancreatic cancer cell line CFPAC1 derived from a liver metastasis [40]. After being transplanted into zebrafish larva, CFPAC1, similar to AsPC1, recruited fewer innate immune cells to the TME and induced the co-localization between innate immune cells and tumors significantly lower than PANC1(Figure 5A,B). By comparing the relative tumor size at 4 dpi to 1 dpi, we found that CFPAC1 also grew significantly larger than PANC1(Figure 5A,C). Moreover, L-clodronate-mediated macrophage depletion led to a similar decrease of tumor size for CFPAC1 in zebrafish xenografts (Figure 5D,E). These results suggest that CFPAC1 also evaded innate immune attack in zebrafish xenografts and molded innate immune cells to support tumor growth.

### 2.6. Knockdown of REG4 in Cancer Cells Activates Innate Immune Responses and Suppresses Engrafted Tumor Growth

The regenerating gene family member 4 (REG4), a small, secreted lectin-like protein, is expressed at a higher level in pancreatic cancer tissues than in adjacent normal tissues. The overexpression of REG4 is related to enhanced pancreatic cancer growth [41,42]. Moreover, REG4 protein or conditioned medium promotes M2 polarization in cultured macrophages and supports cancer cell proliferation in vitro [43]. Inhibition of REG4 cells in pancreatic cells reduced their ability to induce M2 polarization. Therefore, we tested if REG4 is involved in pancreatic-induced innate immune modulation in zebrafish xenografts.

**Figure 6:**
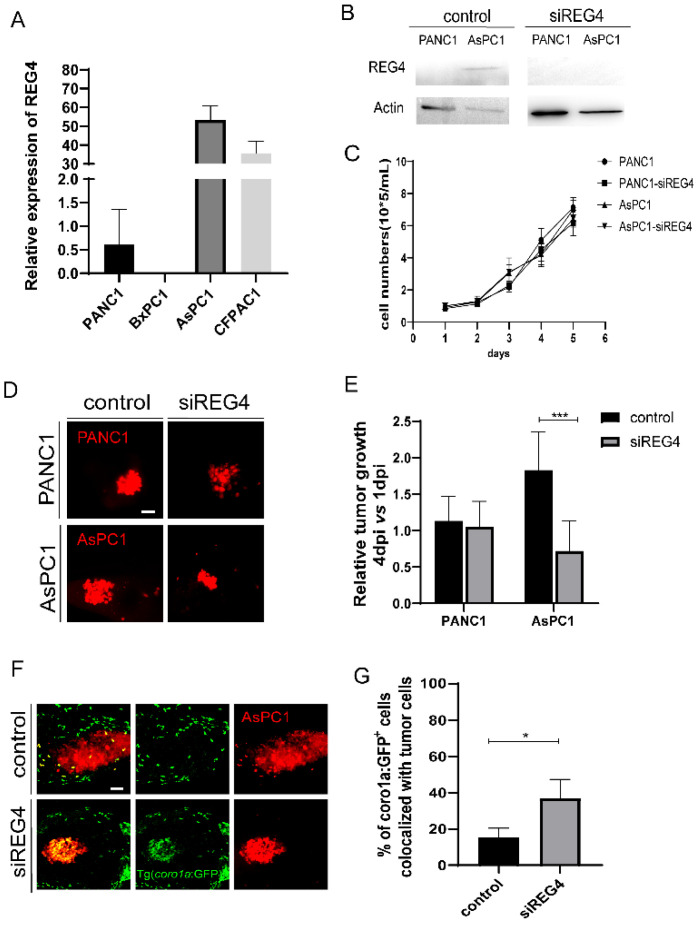
Knockdown of REG4 in cancer cells suppresses tumor growth and activates innate immune response in zebrafish. (**A**) Relative expression of REG4 mRNA in PANC1, BxPC3, AsPC1, and CFPAC1 cells. (**B**) Western Blotting of REG4 and Actin in PANC1 and AsPC1 cells transfected with the control or REG4 siRNA. (**C**) The growth curve of in-vitro culture of PANC1 and AsPC1 cells transfected with the control or REG4 siRNA. (**D**) Representative confocal images of PANC1 and AsPC1 cells transfected with the control or REG4 siRNA in zebrafish xenografts at 4 dpi. (**E**) Relative tumor growth (4 dpi vs. 1 dpi) of the control tumor cells or cells with REG4 silencing. Results are shown as means ± SEM from nine different individuals (*** *p* < 0.001, *t* test). (**F**) Representative confocal images of ASPC1 cells in the control or REG4-silencing group in Tg(*coro1a: GFP*^+^) zebrafish. (**G**) Quantification *coro1a: GFP*^+^ innate immune cell percentage in AsPC1 tumor in the control and REG4-silencing groups. Results are from nine different individuals (* *p* < 0.05, *t* test). dpi, days post injection. Scale bars, 50 µm.

We first examined the expression of REG4 in PANC1, BxPC3, AsPC1, and CFPAC1 cells. The mRNA expression level of REG4 was high in AsPC1 and CFPAC1 cells. In contrast, PANC1 and BxPC3 cells had a low expression of REG4 (Figure 6A). Western blot analysis also confirmed the differential expression of REG4 proteins in PANC1 and AsPC1 cells (Figure 6B). To examine the effects of REG4 in tumor growth, REG4 in PANC1 and AsPC1 cells were knocked down by a siRNA. Inhibition of REG4 did not affect the proliferation of PANC1 and AsPC1 cells in vitro (Figure 6B,C). In zebrafish xenografted embryos, downregulation of REG4 in PANC1 cells did not affect the tumor growth. In clear contrast, the tumor size of AsPC1 cells at 4 dpi was significantly reduced by REG4 siRNA compared with the control (Figure 6D,E). In addition, inhibition of REG4 in AsPC1 cells significantly increased the recruitment and the co-localization of immune cells with the tumors (Figure 6F,G), indicating the activation of the innate immune cells. In addition, we used a second siRNA to inhibit REG4 and observed similar suppression of AsPC1 growth in xenografts (data now shown). These data suggest that like its function to promote M2 macrophage polarization in vitro, cancer-derived REG4 induced innate immune cells to a pro-tumoral state and accelerated the tumor growth in zebrafish xenografts. These data also support that REG4 is a beneficial target for PDAC therapy.

The above results together validate the utilization of zebrafish xenograft model for studying cancer-innate immune cell interaction. Next, we tested if this model could be used to examine innate immune responses to chemotherapy that is known to affect the tumor microenvironment [44]. To do that, we treated the zebrafish xenografts with irinotecan and examined its effects on the interaction between cancer and innate cells. Irinotecan treatment significantly inhibited tumor growth and also suppressed innate immune cells as previously suggested [45]. However, at every dose we tested, we noticed that irinotecan led to a strong reduction of recruitment and co-localization of innate immune cells to the tumor for both PANC1 and AsPC1 (Figure S4C). These results suggest that the remaining tumor-resident innate immune cells after irinotecan treatment were likely at inactivation state. Thus, our studies suggest that irinotecan treatment may elicit an immunosuppressive effect in the TME.

## 3. Discussion

PDAC, a frequent type of pancreatic cancer, remains one of the most challenging problems in clinical fields. The PDAC TME, characterized by high infiltration of myeloid innate immune cells, plays important roles in tumor progression and drug resistance. Recent immunotherapeutic approaches have focused on the adaptive immune system. However, the innate immune system is important for PDAC progression and also suppresses T-cell function and hampers immunotherapy [26]. Hence, there is a need to study how PDAC molds immune cells states and the related molecular mechanism. In this study, we used the zebrafish xenograft model to study the interaction of innate immune cells and pancreatic cancer cells that may present a suitable addition to traditional rodent model systems.

The xenograft and transgenic mouse models are widely used to study immune–tumor cell interactions despite some limitations. Recent developments in biomedical research calling for implementation of the 3-R principles (animal replacement, refinement, and reduction) require alternative models [46]. As an excellent alternative, zebrafish xenograft model for cancer research has gained popularity. Previous studies have successfully used this tool to assess tumor cell proliferation and dissemination as well as screen anti-cancer agents. Here, we used this model to study the crosstalk between the pancreatic cancer cells and the innate immune system. We xenografted four different pancreatic cancer cells and compared their immunogenicity in zebrafish. Our results reveal that cancer cells from the primary tumor (PANC1 and BxPC3) induce anti-tumoral responses of the innate immune cells in zebrafish xenografts. In contrast, cancer cells from metastasis (AsPC1 and CFPAC1) are poorly antigenic and rapidly mold the innate immune cells to pro-tumoral state to fuel tumor growth. Thus, zebrafish can recognize the innate immune cues and reconstitute a distinct innate microenvironment according to different cancers in just 4 days. In our studies, we quantified the tumor size by fluorescent microscopy, which is the standard approach in the field [47,48,49]. To better characterize the tumor growth and the effects of innate immune cells on tumors, tumor cell proliferation and apoptosis by methods such as Ki-67 antibody staining and terminal deoxynucleotide transferase dUTP nick end labeling (TUNEL) require further study. Nonetheless, our results support the zebrafish xenograft as a valid and fast tool to study the innate immune responses inducted by the pancreatic cancer. In addition, our data lay the foundation for the application of the zebrafish model for direct primary tumor transplantation, also known as patient-derived xenografts (PDX), which will help evaluate the cancer immunogenic subtype and select the patients who might be sensitive to immunotherapy. 

At the early stage of zebrafish larvae development, macrophages and neutrophils are two major populations in the innate immune system. Both macrophages and neutrophils have been shown to regulate anti-tumor immunity and immune evasion [9]. In our research, PANC1 and BxPC3 recruited more *coro1a: GFP^+^* innate immune cells to the TME compared to AsPC1 cells, and the co-localization of *coro1a: GFP*^+^ cells with the tumor cells was increased from 1 dpi to 4 dpi by three-fold for PANC1 and BxPC3. In contrast, PANC1, BxPC3, and AsPC1 recruited a comparable number of *mpx:GFP^+^* neutrophils to the tumor area, and the co-localization of *mpx:GFP* with tumor cells did not change from 1 dpi to 4 dpi for all three cells. Thus, we reasoned that macrophages might be the major populations in *coro1a: GFP^+^* innate immune cells recruited in the TME area. We used Tg(*mpeg1: GFP*) to visualize macrophages, but did not detect good fluorescent signal. Future studies using other reporter lines, such as Tg (*mpeg1: mCherry-F*), to label macrophages as previously suggested [26], might help verify the responses of macrophages in the PDAC TME. Functionally, we showed that pharmacological deletion of macrophages [18,26] significantly reduced AsPC1 and CFPAC1 growth. In addition, AsPC1 xenografted in zebrafish *irf8^−/−^* larva, which have decreased macrophages but increased neutrophils, also showed significant decreased tumor growth. All these data together suggest that macrophages might be more sensitive to the immune cues and play more important roles in our system. Our results are consistent with the idea that macrophages are the driver of innate immune states in the TME [33]. 

Macrophages M2 polarization is largely responsible for the innate immune pro-tumoral state and the immune suppression in the TME. Inhibiting M2 polarization or reprograming macrophages may hold the key to immune activation and anti-tumor response. Mounting evidence indicates that the tumor cells educate and reprogram the TAMs towards M2-like phenotypes. In PDAC, factors that are involved in M2 polarization and the innate immune suppression are poorly defined [50,51]. Previous studies show that pancreatic cancer-derived factor REG4 promotes cultured macrophage to a M2 phenotype and stimulates cancer cell proliferation in vitro [43]. Here, we demonstrate that AsPC1 cells express high level of REG4, and that inhibiting REG4 in AsPC1 cells, when xenografted in zebrafish, activates innate immune cells and strongly suppresses the tumor growth. Thus, REG4 plays a role in innate immune suppression. REG4 may also induce macrophages M2 polarization in zebrafish. Future studies using double transgenic line Tg(*mpeg1:mCherry-F;tnfa:eGFP-F*) that could distinguish M1 and M2-like macrophages [52] will help evaluate the specific polarization and function of macrophages in zebrafish xenografts. In addition, future studies should also examine the innate immune response and tumor growth of PANC1 cells with REG4 overexpression, the results of which will reveal if REG4 is sufficient to induce innate immune suppression in zebrafish and further verify its function in pancreatic cancer-innate immune interaction and tumor progression. Our results, together with the results from previous in vitro studies, support that targeting REG4 may offer therapeutic benefits for PDAC. Our data also support that zebrafish xenograft is a valid model to study the molecular mechanism underlying in-vivo innate immune modification by pancreatic cancer. We found that cancer cells from metastasis more likely induce a pro-tumoral state of innate cells, which emphasizes the need for a better understanding of the molecular and cellular characteristics in the metastatic cancers and their tumor microenvironment. We propose that by alternating signaling in cancer cells and/or using the wild-type and mutant zebrafish together with different transgenic lines, it is possible to dissect the molecular mechanism underlying PDAC-educated innate immune modification, particularly for neutrophils and macrophages. Data generated from these assays will contribute to a better understanding of the interaction between the tumor and the tumor innate immune microenvironment. Thus, zebrafish xenograft model of pancreatic cancer will be useful to identify novel targets in innate immune activation and hence enhance immunotherapy. 

In summary, we show that zebrafish xenograft of pancreatic cancer is a fast and reliable system sensitive to cancer cell immunogenicity. It provides an intuitive in-vivo model to study pancreatic cancer-mediated modulation of innate immune cells, the underlying molecular mechanism, and the function of instructed innate immune cells, particularly macrophages, during tumor progression.

## 4. Materials and Methods

### 4.1. Pancreatic Cancer Cell Lines and Culture

Human pancreatic cancer cell lines PANC1, BxPC3, AsPC1, and CFPAC1 were purchased from the Shanghai Institute of Biochemistry and Cell Biology, Chinese Academy of Sciences. PANC1, BxPC3, and CFPAC1 were maintained in Dulbecco’s modified Eagle’s medium (DMEM, Gibco, Carlsbad, CA, USA) supplemented with 10% fetal bovine serum (FBS, Gibco, Carlsbad, CA, USA). AsPC1 were maintained in Roswell Park Memorial Institute 1640 (RPMI 1640, Gibco, Carlsbad, CA, USA) supplemented with 10% FBS. All cells were cultured in 5% CO_2_ atmosphere at 37 °C.

### 4.2. Zebrafish Maintenance and Embryo Handing

Zebrafish were maintained, handled, and bred according to standard protocols from the Institutional Animal Care Committee of Shanghai Jiao Tong University. Adult zebrafish were kept at 26–28 °C and the light-dark cycle was 14 h:10 h. Embryos were obtained by crossing one male and two females in tanks and kept at 28.5 °C in E3 medium (5 mM NaCl, 0.17 mM KCl, 0.33 mM CaCl_2_, and 0.33 mM MgSO_4_). Embryos were staged by hours post-fertilization (hpf) and days post-fertilization (dpf). Tg(*coro1a: GFP*) zebrafish and *irf8^−/−^* mutants were kindly provided by Professor Li Li from Southwest University, China. Wild type AB strain, Tg(*mpx:GFP*) zebrafish were obtained from China zebrafish resource center.

### 4.3. In Vitro Cell Proliferation Analysis

For cell proliferation, PANC1, BxPC3, and AsPC1 (5 × 10^4^ cells/well) were plated in three 48-well plates independently and incubated for 5 days at 37 °C with 5% CO_2_. Each day, trypsin-EDTA (Sigma-Aldrich, St. Louis, MI, USA) were added into three wells to digest cells respectively in three 48-well plates, and the number in each well was obtained by counting on a cell counter.

### 4.4. In Vitro Cell Viability Assay

Cell viability was measured by 3-(4,5-dimethylthiazol-2-yl)-2,5-diphenyltetrazolium bromide (MTT) assay (Beyotime Biotechnology Corporation Ltd., Shanghai, China). PANC1, BxPC3, and AsPC1 (5 × 10^3^ cells/well) were cultured in 96-well plates respectively and incubated for 24 h. Then PANC1, BxPC3, and AsPC1 were exposed to irinotecan (2, 4, 8, 20, 40, 60, 80, 100, 250, and 500 μM). The control group was treated with the same volume of DMSO. After 72 h of incubation, a 150 μL MTT solution (0.5 mg/mL 3-(4,5-dimethylthiazol-2-yl)-2,5-diphenyltetrazolium bromide in phosphate-buffered saline) was added into each well and incubated for 4 h. Then, the supernatant fluid was removed and 150 μL dimethyl sulfoxide (DMSO) was added per well. Optical density was read at 490 nm on a microplate reader.

### 4.5. Cell Staining and Zebrafish Xenografts

PANC1, BxPC3, and AsPC1 were labeled with Dil (Invitrogen, Carlsbad, CA, USA) for 15 min at 37 °C and then 20 min at 4 °C. Labeled cells were washed in 100% FBS then in 67% DPBS twice, finally resuspended in 4% PVP-K30 (Polyvinylpyrrolidone K30). The final cell concentration is 1 × 10^7^ cells/mL. Trypan blue staining was used to assess cell viability. The cell viabilities of PANC1, BxPC3, and AsPC1 were all higher than 95% before transplantation. Two dpf zebrafish embryos were anesthetized with 0.02% tricaine (Sigma-Aldrich, St. Louis, MI, USA) and positioned into the left side on a wet agarose pad. Approximately 500–700 cells were injected into yolk of zebrafish embryo using borosilicate glass microcapillaries under SZX16 stereomicroscope (Olympus, Tokyo, Japan). After maintaining at 32 °C for 2 h post injection, embryos with the absence of tumor cells at yolk sac or with tumor cells in the circulation system were discarded. Embryos were then kept at 34 °C to the end of experiments. Xenografts were checked on a daily basis, and the E3 medium was refreshed daily. The dead and non-xenografted ones were removed once identified.

### 4.6. Imaging and Analysis of Zebrfish Xenografts 

Zebrafish xenografts were anesthetized with tricaine and buried in 0.8% low-melt agarose (Shuhong Biotechnological Corporation Ltd., Shanghai, China) in plates and then imaged under a confocal microscope Leica SP8 microsystems (Leica, Heidelberg, DE, Germany) with a 5 μm interval in a total of ~100 μm stack using the Z-stack function [27]. Animals in the same experiments were imaged under the same conditions, and the tumor size was quantified by measuring the area and fluorescence intensity of 2D image with ImageJ software according to previous studies [47,48,49]. The tumor growth was evaluated at 1 dpi and 4 dpi, and the relative tumor growth was calculated as the ratio of the tumor size at 4 dpi to 1 dpi, similar to previous reported methods used in [26,27,48]. The number of green *coro1a: GFP^+^* cells, *mpx:GFP^+^* cells, or Dil-labelled tumor cells in the images acquired by maximum intensity projection were qualified using ImageJ software Cell counter plugin. 

### 4.7. Drug Treatment of Zebrafish Xenografts

Pancreatic xenografted zebrafish were randomly transferred to 24-well plates (15 embryos/well) at 1 day post injection (1 dpi). Drugs were added directly into E3 medium at the final concentration of 50 μM for irinotecan, 150 μg/mL for LPS, and 40 μM for PTX. The control groups were maintained in E3 medium with 0.1% DMSO. Xenografts were checked on a daily basis to remove dead ones, drugs were refreshed daily, and the treatment continued for 3 days.

### 4.8. Zebrafish Macrophage Ablation with Clodronate Liposomes

Liposomes-encapsulated PBS (L-PBS, Yeasen Biotech Corporation Ltd., Shanghai, China) and liposomes-encapsulated clodronate (L-Clodronate, Yeasen Biotech Corporation Ltd., Shanghai, China) were used to deplete macrophages in embryos. Cells were re-suspended in PBS, L-PBS, or L-Clodronate respectively at a final concentration of 1 × 10^7^ cells/mL before being injected into the yolk of zebrafish larva.

### 4.9. Flow Cytometry and Cell Sorting

At 4 dpi, PACN1, BxPC3, and AsPC1 xenografts in Tg(*coro1a: GFP*) background were anesthetized and washed in PBS three times. The xenografts were then incubated at 37 °C with 38 μg/mL of Liberase (Roche, Mannheim, Germany). Twenty minutes later, 10% FBS was added to stop the reaction, followed by filtration (40 µm filter) and centrifugation (1500 rpm, 4 °C, 10 min). The resulting single cells were resuspended with 500 µL PBS plus 10% FBS. GFP positive cells were sorted with FACS Aria III (Becton, Dickinson and Company, San Jose, CA, USA) and collected in PBS buffer.

### 4.10. Gene Expression by Real-Time qPCR

The sorted *coro1a: GFP^+^* cells were lysed in TRIzol LS reagent (Thermo Fisher Scientific, Waltham, MA, USA), and total RNA was extracted according to the manufacture’s protocol. cDNAs were synthesized from total RNA using the HifairⅢ 1st Strand cDNA Synthesis SuperMix reagent Kit (Yeasen, Shanghai, China). Hieff qPCR SYBR Green Master Mix (Yeasen, Shanghai, China) was used for qPCR analysis. The relative expression of targeted genes was calculated using the 2^−ΔΔCT^ method. The primers for IL-10, IL-12, and TNF-α are listed in Table 1.

### 4.11. Transfection of siRNA

PANC1 or AsPC1 cells at a concentration of 1 × 10^5^ cells/mL were plated into a 6-well dish. After 24 h of incubation, culture medium in each well were removed and replaced with 2 mL of fresh culture medium. Each well was transfected with 2.5 μg siRNA in Opti-MEM free of serum with 5 μL Lipo6000 (Beyotime, Shanghai, China) for 6 h, then the transfection medium was removed and the fresh culture medium was added. The cells were incubated for 3 days before analysis or transplantation. The sense sequence of REG4 siRNA was: GAUAUCAUCAUGAGACCCATT, andthe antisense of REG4 siRNA was: UGGGUCUCAUGAUGAUAUCTT. The sense sequence of the second REG4 siRNA was: GGCCAUGUAUCUGUACAGATT, andhe antisense of the second REG4 siRNA was: UCUGUACAGAUACAUGGCCTT (Shanghai GenePharma Co., Ltd., Shanghai, China).

### 4.12. Western Blotting

Cells at a concentration of 1 × 10^5^ cells/mL were plated into a 6-well dish. After 3 days of incubation, cells in each well were lysed with lysis buffer at 4 °C for 30 min. The lysates were loaded and analyzed using SDS-PAGE following standard protocols. Primary antibodies used were anti-REG4 antibody (1:500, Abcam, Cambridge, UK) and anti-Actin antibody (1:1000, Proteintech, Wuhan, China). Secondary antibodies used were anti-mouse antibodies (1:1000, Proteintech, Wuhan, China) and anti-rabbit antibodies (1:1000, Proteintech, Wuhan, China).

### 4.13. Statistical Analysis

GraphPad Prism 8.0.2 (GraphPad Software, Inc.: San Diego, CA, USA, 2019, https://www.graphpad.com, accessed on 21 March 2021) was used to analyze all data. The values of all triplicate experiments are presented as mean ± SEM. The statistical significance was displayed as “ns” for no statistical significance, “*” for *p* < 0.05, “**” for *p* < 0.01, “***” for *p* < 0.001, and “****” for *p* < 0.0001.

## Figures and Tables

**Table 1:** Real-time qPCR primer sequence.

Primer	Nucleotide Sequence 5′-3′
IL-10 F	TCACGTCATGAACGAGATCC
IL-10 R	CCTCTTGCATTTCACCATATCC
IL-12 F	GAAACTCAACTGACCTCAACTG
IL-12 R	CTTTATCTGGCTTGACAATGTCTC
TNF-α F	GCGCTTTTCTGAATCC TACG
TNF-α R	GATCACCTGTGTGCTCATCG

## Data Availability

The data presented in this study are available in the article and Supplementary Material.

## Supplementary Materials

The following supporting information can be downloaded at: https://www.mdpi.com/article/10.3390/ijms23126442/s1.
