# Supplementary material for: Zebrafish Xenograft Model for Studying Pancreatic Cancer-Instructed Innate Immune Microenvironment
Source: Int J Mol Sci. 2022 Jun 9;23(12):6442. doi: 10.3390/ijms23126442 (PMC9224329; doi:10.3390/ijms23126442)
Supplement: Supplementary file 1 [file ijms-23-06442-s001.zip › ijms-1709974-supplementary.pdf]

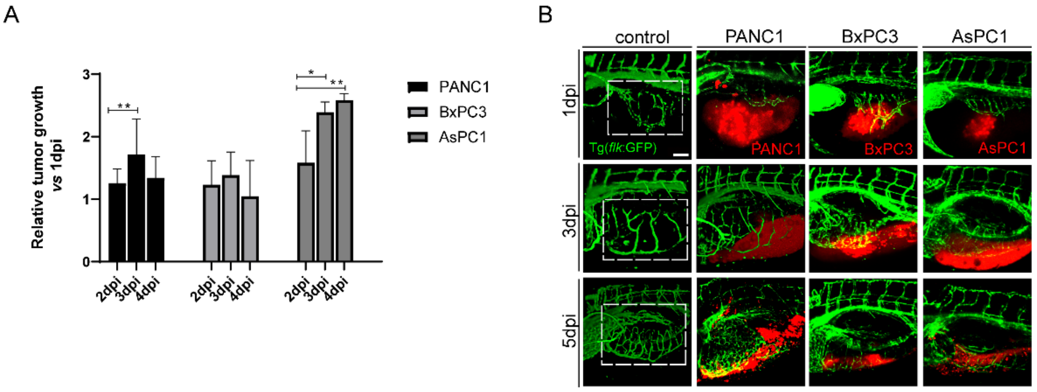

Figure S1. The daily growth and angiogenesis of PANC1, BxPC3, AsPC1 cells in zebrafish xenografts. (A)Relative tumor growth for PANC1, BxPC3, AsPC1 xenografts at 2-4 dpi vs 1dpi. Results are shown as means ± SEM from 9 different individuals (*p< 0.05, **P<0.01, t test). (B) Representative confocal images of the sub-intestinal vessels in PANC1, BxPC1, AsPC1 xenografts at 4 dpi in Tg(flk:GFP) background. The white boxes in the left column mark the sub-intestinal vessels and are magnified in the right three columns. dpi, days post injection. Scale bar, 50 μm.

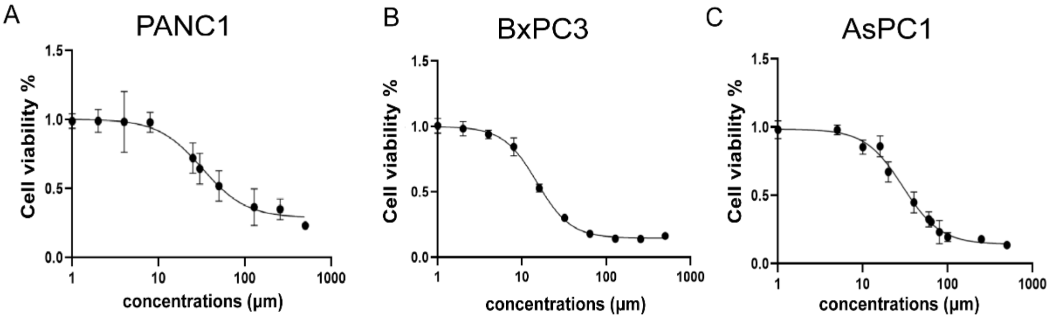

Figure S2. Dose-effect curve of irinotecan in PANC1, BxPC3, AsPC1 cultured cells.

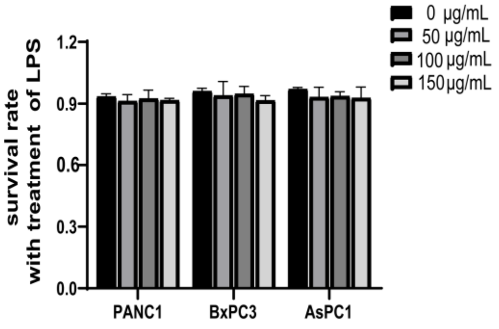

Figure S3. The survival rate of *in vitro* cultured PANC, BxPC3, AsPC1 cells after the treatment of LPS at different concentrations.

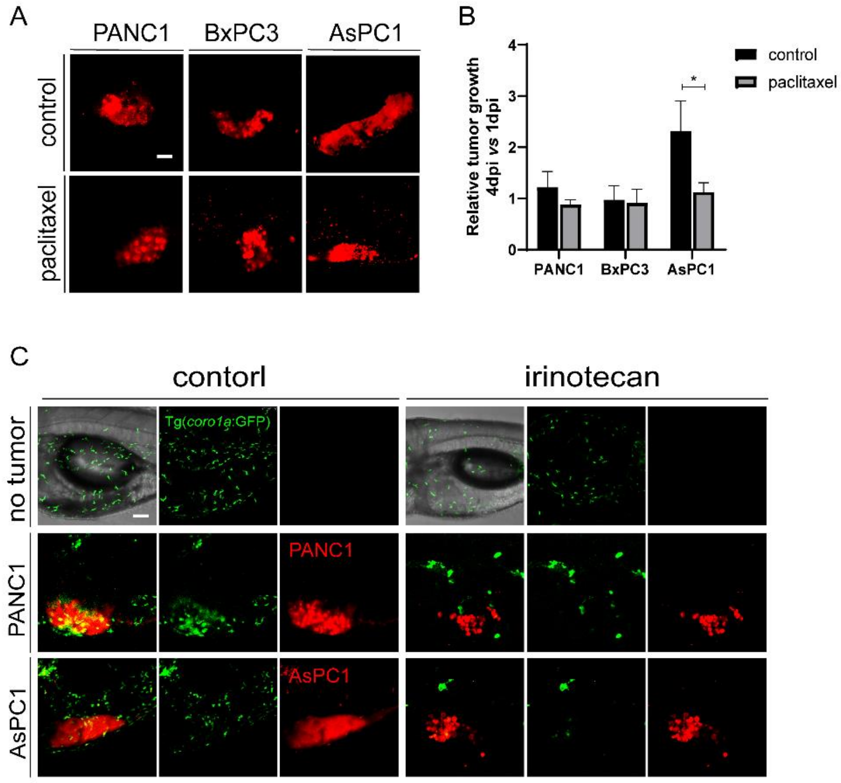

Figure S4. The effects of paclitaxel and irinotecan to pancreatic cancer progression. (A)Representative confocal images of PANC1, BxPC3, AsPC1 xenografts at 4 dpi after the treatment of DMSO (control) or paclitaxel (40 μM) respectively. (B) Relative tumor growth in xenografts after treated with DMSO or paclitaxel. Results are shown as means ± SEM from 9 different individuals (*p< 0.05, t test). (C)Representative confocal images of PANC1, AsPC1 xenografts in Tg(coro1a: GFP) at 4dpi after the treatment of DMSO (control) or irinotecan (50 μM). dpi, days post injection. Scale bars in A and C, 50 μm.
